# Treatment of Intrusive Suicidal Imagery Using Eye Movements

**DOI:** 10.3390/ijerph14070714

**Published:** 2017-06-30

**Authors:** Jaël S. van Bentum, Marit Sijbrandij, Marcus J. H. Huibers, Annemiek Huisman, Arnoud Arntz, Emily A. Holmes, Ad J. F. M. Kerkhof

**Affiliations:** 1 Department of Clinical Psychology, Vrije Universiteit Amsterdam (VU), van der Boechorststraat 1, 1081 BT Amsterdam, The Netherlands; e.m.sijbrandij@vu.nl (M.S.); m.j.h.huibers@vu.nl (M.J.H.H.); a.huisman@vu.nl (A.H.); ajfm.kerkhof@vu.nl (A.J.F.M.K.); 2 Department Psychology, University of Pennsylvania, Stephen A. Levin Building, 425 S. University Ave, Philadelphia, PA 19104-6018, USA; 3 Faculty of Behavioural and Social Sciences, Youth as a Social Phenomenon–Youth Studies, University of Groningen, Grote Rozenstraat 38, 9712 TJ Groningen, The Netherlands; a.huisman-geleijnse@rug.nl; 4 Department of Clinical Psychology, Universiteit van Amsterdam, Nieuwe Achtergracht 129, 1018 WS Amsterdam, The Netherlands; A.R.Arntz@uva.nl; 5 Division of Psychology, Department of Clinical Neuroscience, Karolinska Institutet, SE-171 77 Stockholm, Sweden; emily.holmes@ki.se

**Keywords:** suicide, suicidal mental imagery, flash-forwards, intrusions, preventive intervention, eye movement dual task (EMDT)

## Abstract

Suicide and suicidal behavior are major public health concerns, and affect 3–9% of the population worldwide. Despite increased efforts for national suicide prevention strategies, there are still few effective interventions available for reducing suicide risk. In this article, we describe various theoretical approaches for suicide ideation and behavior, and propose to examine the possible effectiveness of a new and innovative preventive strategy. A model of suicidal intrusion (mental imagery related to suicide, also referred to as suicidal flash-forwards) is presented describing one of the assumed mechanisms in the etiology of suicide and the mechanism of therapeutic change. We provide a brief rationale for an Eye Movement Dual Task (EMDT) treatment for suicidal intrusions, describing techniques that can be used to target these suicidal mental images and thoughts to reduce overall behavior. Based on the available empirical evidence for the mechanisms of suicidal intrusions, this approach appears to be a promising new treatment to prevent suicidal behavior as it potentially targets one of the linking pins between suicidal ideation and suicidal actions.

## 1. Introduction

Suicidal ideation and behaviors are major public health problems and have been estimated to affect 3–9% of the population worldwide [1]. In the Netherlands, a total of 1871 suicides occurred in 2015, compared to 1353 suicides in 2007 [2]. The standardized (based on age distribution) suicide rate shows that while 2007 saw an average of 8.3 suicides per 100 thousand inhabitants, by 2015 this had increased to 11.1 [2]. This indicates that, despite the many efforts to prevent suicide, suicide rates are still rising. Several interventions have been developed to prevent suicide [3], but it is unclear which processes link suicidal plans to the act of suicide [4]. If the psychological processes underlying both suicidal ideation and the decision to act on suicidal thoughts are understood, it could help develop specific interventions to target suicidal ideation when it first emerges and prevent the transition to a suicide attempt [4]. Recent studies provide a fresh perspective on suicide prevention.

In the passage from having suicidal thoughts and actual suicidal behavior, suicidal patients may develop a cognitive narrative to reflect on suicide [5,6]. This narrative, also called rumination, is defined as ‘compulsively focused attention on the symptoms of one’s distress and on its possible causes and consequences, as opposed to its solutions’ [7]. Repeated attention to problems and activation of negatively biased thoughts in response to these problems may intensify the impact of a negative life event [8]. In addition to this cognitive narrative, suicidal individuals may form mental images of how to complete suicide [5,6]. These mental images (e.g., imagining a suicidal act, or one’s own funeral) have also been referred to as ‘flash-forwards’ [5,9,10,11]. In comparison to suicidal verbal thoughts, which are language-like descriptive formats, these suicidal mental images appear through picture-like depictive formats. Thus, while the content of verbal thoughts and mental images in this case revolve around suicide, the format used to represent the information differs [12]. In addition, experimental research suggests that imagery more readily elicits an emotional response than verbal information of the same content [13]. Suicidal imagery may then evoke feelings of distress more powerfully than would be the case if it remained in a verbal form [13,14].

Given that suicide-related images and thoughts are conceptually linked to the process of rumination, suicidal mental imagery may play a pivotal causal role in the development of suicidality [15]. Extensive evidence shows a clear association between intrusions and rumination [16,17,18,19]. In this article, intrusions refer to uncontrollable and involuntary mental imagery related to suicide [5,7,8,9]. Ehlers and Clark [20] suggest that rumination may intensify negative emotions, which in turn create internal cues to trigger intrusions. In fact, an increasing number of studies have shown that patients diagnosed with unipolar depression [5,9] or bipolar disorder [10,11] report repetitive intrusive suicide-related images and thoughts. Although we focus on affective disorders, suicidal images may be relevant in other disorders as well, such as anxiety disorders [13,21]. These suicidal images may be both distressing and comforting [13,22], and can be seen as a last visualized escape to protect oneself against future adversity [22,23]. Examples include imagining oneself during or right after a suicide attempt (taking an overdose, jumping off a bridge), or visualizing the reaction of family members. As the frequency (amount of occurrences) increases, these intrusive suicidal images can be experienced as more and more adverse and inescapable, and in the end may become intolerable. Finally, dying by suicide may feel like the final and only way to escape this intrusive imagery, and the individual protects him or herself from an unendurable misery and prolonged exposure to these images [22]. Although clinicians are aware of the existence of suicidal imagery, it is unusual to inquire about them, or to treat these images as a focal point in the intervention [24].

Cognitive psychology suggests that imagining oneself in a potential future action affects the subsequent behavior [5,25,26]. In fact, Carrol [27] advocates that imagining an event increases the likelihood of engaging in that event as well as the perceived event probability. This leads to the proposition that, if imagining a future event is causally implicated in actual behavior, engagement with suicide-related future oriented imagery may prompt suicidal behaviors [5,26,28]. Imagery may be a particularly useful target in this context, since it is regarded as an as-if simulation of reality and to promote action [29]. Our central assumption is that the frequency and intrusiveness of suicidal imagery impacts the actual risk of suicide. We hypothesize that the more frequently the suicidal intrusive images appear, the more discomfort is experienced. Likewise, the more involuntarily and uncontrollably (intrusive) these images appear, the more discomfort and distress will be experienced. We suggest that the pathway from suicidal ideation to suicidal behavior simulates obsessive-compulsive symptomatology in that an increased presence of intrusive suicidal imagery leads to increased distress, which in turn leads to suicidal ideation and finally suicidal behavior. In addition, these uncontrollable images may indirectly function as a repeated imaginal exposure to the actual behavior, making the transition from thoughts to behavior potentially more readily available.

As mentioned previously, the intrusive nature of these suicidal images increases their discomfort and potential danger, i.e., that they spring to mind involuntarily. Intrusive suicidal ideas and images are predominantly experienced as unwelcome, burdensome, involuntary, and are difficult to manage or eliminate [5,14]. In addition to its uncontrollable nature, the emotionality and vividness can increase the discomfort of a particular intrusion. Trauma research evidence shows that intrusive, traumatic images are causally related to distress, as they can be persistently re-experienced with the original emotions and sensations (i.e., trauma and flashbacks in Posttraumatic Stress Disorder (PTSD)) [30]. In this article we make use of various theoretical models, like the Escape Theory [31] and the Interpersonal-Psychological Theory [32] to underpin the concept of suicidal intrusions and our central proposition. Furthermore, we hypothesize that an intervention directly targeting these intrusive suicidal thoughts and images to reduce their frequency of occurrence may directly decrease the risk of suicidal behavior of an individual. Experimental studies have shown that vividness and emotionality of negative autobiographical memories is reduced when individuals retrieve the memory, and perform a dual task simultaneously that taxes the working memory, such as eye movements or counting backward [33,34]. This is also one of the various presumed working mechanisms of the evidence-based treatment Eye Movement Desensitization and Reprocessing (EMDR) for PTSD. We believe that these suicidal intrusions may appear as mental images or verbal thoughts, or both. While we want to target these suicidal intrusions altogether, our explicit focus is on performing eye movements when retrieving a mental suicide-related image, since dual tasks are assumed to target especially images [35]. Therefore, we propose an Eye Movement Dual Task (EMDT) intervention that taxes working memory while retrieving images as an add-on intervention to usual care for suicidal patients.

## 2. Theoretical Background of Suicidal Intrusions

The following quote depicts a clinical example of a suicidal intrusion. It portrays the existence of these suicidal intrusions in a clinical setting.

Miss A. 37 years of age, continuously had thoughts and images about killing herself during the last 4 years. She had vivid images of herself jumping in front of a train, seeing her body lying disfiguredly alongside the tracks, thoughts and visions of her funeral, and the sadness of her parents. Immediately thereafter she had thoughts and images of herself slaying her wrists and bleeding to death, followed by vivid fantasies of herself hanging from a rope. She tried intensively to stop these images, but she could not stop it, and she could not sleep at all. She was exhausted and hated herself. She said that she actually did not want to kill herself, but because of the obsessive nature of her intrusions, she finally wanted to escape her consciousness and requested assisted suicide. She tried all medications and other treatments made available to her by the clinic where she was under mental health care treatment. She was then treated with Cognitive Behavioral Therapy (CBT) intervention aimed at obsessive rumination and repetitive imaginations (worry postponement and exposure) and which contributed significantly to her recovery. The total time of suicidal intrusive rumination was reduced from 20 h a day to 45 min a day and Miss. A. withdrew her request for assisted suicide. More information about this real-life story can be found in Kerkhof and Van Luijn, Suicidepreventie in de praktijk.

*[36]* *(pp. 104–107)*

There has been extensive research on intrusions. Evidence shows that the majority of the population in a non-clinical sample experiences intrusive thoughts (79–99%) and do not view them as problematic [37]. However, the appraisal of the uncontrollable thoughts is what creates the problematic experience in some individuals. The cognitive behavioral model [38] implies that when interpreted as being personally significant and meaningful, the intrusive thought may become obsessive in nature. Berntsen [39] argues ‘intrusions are experienced as involuntary and uncontrollable in that their appearance in consciousness is spontaneous rather than following a deliberate effort or search’ [40]. While intrusions can possess a multitude of sensory qualities (visual, auditory, olfactory, touch, and movement), most literature is concerned with visual images [40]. Moreover, growing evidence supports the existence of intrusive suicidal ideas and images [5,9,10,11,41]. Intrusive mental images seem to be an important feature in multiple psychological disorders, like PTSD [30,42], agoraphobia [43], and social phobia [44] (see Holmes and Mathews [21] for a review). Disorders differ in intrusions due to their associated theme, as the content of the intrusions matches the specific content of verbal thoughts related to each disorder [40]. Research on memories shows that their intrusiveness is supposed to be related to the intensity rather than the valence of the memory [39]. According to the memory-based model, there is one memory system but the retrieval of memories differs for involuntary and voluntary memories [45,46,47]. While voluntary memories are retrieved using controlled narrative and schema-based searches, involuntary memories are retrieved by an uncontrolled associative spreading activation. This means that a cue alone is sufficient to prompt retrieval of this particular involuntary memory [40]. It allows for less emotional regulation, leading to a more emotional reaction and mood change compared to voluntary memories [48]. In addition, a recent study suggest that depressogenic intrusions may ‘reflect the effects of high levels of emotion on the entire memory system rather than any qualitative alteration in the way the system is operating’ [40].

In the pre-suicidal process, intrusive thinking and intrusive imagery are often reported. The pre-suicidal process is a period in which an individual gradually gravitates towards the act of suicide, and he or she can become preoccupied with emotional thoughts and images about suicide (i.e., suicidal imagery) [49]. To be able to comprehend suicidal intrusions, we need to evaluate the theoretical background of how the suicidal images come about in a suicidal person. According to Ringel [23] the pre-suicidal syndrome is seen as the endpoint of a specific neurotic development, and instead of neurotic images it leads to suicide. Initially, the person creates a tunnel vision, in which they let go of reality and focus on a fantasy world that becomes obsessive in nature. He or she loses sense of reality, and takes distance from others. This fantasy world includes suicidal thoughts and images and may push them to increasingly fixate on the end of that tunnel: suicide. The initial anger and aggression felt by the individual, perhaps due to negative life events, is no longer directed toward the outside world, but rather turned inwards. Aggression and impulsiveness have been shown to be risk factors for suicide [50,51]. Obsessive fantasies and imagination are focused on the belief that suicide is the only way to prevent worse things from happening. At first, the individual may actively visualize the images, as they provide a hypothetical scenario for a potential escape, punishment or revenge. However, as the images gradually become involuntary and uncontrollable, it may be assumed that they become frightening and alarming over time. Their loss of control about suicide becomes upsetting, and people start trying to avoid them. Recently, empirical studies verified this obsessive imagination and it is now referred to as ‘suicidal mental imagery’ [6,41,52], or flash-forwards to suicide [5,9,10,11]. They include repetitive, intrusive, uncontrollable, vivid images that may occupy the suicidal mind with repetitive ideation, plans, and behavioral intentions. Suicidal individuals report these images as if ‘they are watching a clear and vivid video of their own death by suicide’ [6]. 

The cognitive behavioral model of Salkovskis [38] suggests that unwanted thoughts develop into obsessions when they elicit suppression attempts, which, in turn, result in a paradoxical increase in thought frequency. Abramowitz et al. [53] found that longer suppression periods were related to larger initial enhancement effects, suggesting that efforts to suppress unwanted thoughts become less successful over time. In other words, the cognitive effort of stopping suicidal intrusions appears to have a counterproductive effect and inflates their frequency and intensity instead. This leads to feelings of uncontrollability related to suicidal ideations and images. The uncontrollability in turn may lead to the wish to stop consciousness altogether, prompting the last motivation for suicide: to escape from the unbearable, intrusive ideation and imagery. These assumptions are all closely tied into the Escape Theory proposed by Baumeister [31]. The Escape Theory is an integrative framework that provides a theoretical model to explain the process through which individuals develop suicidal ideation, ‘with a causal chain characterizing suicide as the final step in self-destructive behavior’ [54]. The theory states that suicide emerges as an escalation of the person’s wish to escape from meaningful awareness of current life problems and their implications about the self. Suicide is viewed as an attempt to escape aversive self-awareness.

The Escape Theory can be categorized into a causal chain of six stages, potentially leading to an ultimate suicide act [31]. First, stressful life-events fall severely short of standards or expectations, or both. Second, these failures are attributed internally leading to high painful self-awareness. Third, the individual displays negative affect and experiences negative feelings due to this painful self-awareness. Fourth, trying to escape these feelings of negative affect and self-awareness, the individual enters a state of cognitive deconstruction. This helps to separate the self from meaningful self-awareness or emotions, and the individual now has a constricted temporal focus and solely immediate or proximal goals. Fifth, the cognitive deconstruction leads to the removal of inhibition, passivity, an absence of emotion and an increase in irrational thoughts. Like Ringel’s pre-suicidal syndrome, the irrational thoughts appear in an obsessive manner, and may create a kind of vacuum by removing familiar beliefs and preventing meaningful thought about reality. Sixth, an increase in accessibility to suicidal ideation occurs, and drastic measures, like suicide, become acceptable. The Escape Theory suggests that as the fear of the intrusive, repetitive force of suicidal intrusions increases, the fear to act upon them decreases. Ultimately, the intrusions become more aversive and attractive to act upon. In order to escape the constant chaos in their head created by the unstoppable, vivid suicidal intrusions, suicide seems to be the best (or only?) possible solution as it offers the individual a state of ‘oblivion’.

Recent studies provide initial evidence for the Escape Theory, as it was found that when individuals realize their failure to attain an important standard, they experience increased accessibility of suicidal thoughts [55]. Moreover, individuals with internal Locus of Control (how strongly people believe they have control over situations and experiences) showed greater implicit suicidal mind (i.e., suicidal ideation), when primed with thoughts of failure compared to a control condition. This confirms the assumption of the Escape Theory that failure should lead to suicide only if it is internally attributed [54]. Attributing responsibility for failure to one’s self strains the self and gives a strong desire to escape (cognitive dissociation). Suicide attempters had significantly stronger implicit associations between death and the self than other psychiatrically distressed individuals who did not make an attempt [56]. In close relation to the internal attribution of failures, Joiner’s Theory of Interpersonal-Psychological Theory [32] suggests that when an individual holds two specific psychological states (perceived burdensomeness and low belonging) in their minds, they may develop the desire for death. Perceived burdensomeness refers to the view that one’s existence burdens family, friends, or society, or both, while a low sense of belongingness refers to the experience that one is alienated from others (i.e., family, circle of friends, or other valued groups). The theory proposes that, in addition to the desire to die by suicide, an individual must be able to do so (acquired capability [32]). Repeated exposure to painful of fearsome experiences may result in habituation and a higher tolerance of pain and fearlessness of death. The suicidal intrusions may then work as this increased, repetitive exposure that, in turn, increases the individual’s acquired capability to enact lethal self-injury. Studies have shown that imagining a future event in general increases the likelihood that a person acts upon it [28]. Therefore, we assume that by reducing suicide-related images, we may also decrease the likelihood that a person would engage in suicidal behavior. Thus, suicide-related intrusive images can be crucial targets for suicide prevention.

Clinical studies [57,58] demonstrate correspondence between intrusive images of dreaded future outcomes (i.e., in Obsessive Compulsive Disorder.(OCD) and anxiety disorders) and intrusive memories of past events (i.e., PTSD). Several cognitive theories try to explain the underlying role of memory for intrusive thoughts and traumatic events in PTSD [20,40,59,60,61]. According to the autobiographical memory model [62], the traumatic experiences consist of fragmented sensory details because they have been inadequately incorporated within the contextually organized autobiographical knowledge base. For example, patients with depressive symptoms tend to recall many ‘over-general’ memories, as a constant reminder of their failure [63]. The traumatic experience seems to be stored in the long-term memory with the same emotional intensity as when the event occurred. Interestingly, regular autobiographical memories tend to consist of detailed visual scenes explicitly linked to an original experience, while intrusive images tend to represent an imaginal extension of the original experience [64]. For example, an individual might experience a suicidal intrusion as the successful completion for a previous suicide attempt. The sensory impressions are experienced as if happening in the present, rather than being memories from the past, and the emotions are the same as those experienced at the time of the event [20].

## 3. Treatment of Intrusive Images

Experimental research has indicated that imagery may elicit stronger emotional responses than do corresponding verbal cognitions [13,65], thus making them an important target in interventions. Moreover, intrusions tend to be a main target in disorders that are most typically associated with negative intrusions, such as anxiety disorders and PTSD. Very little clinical work or research to date has focused on how to treat suicidal mental imagery [66] and new initiatives are required.

A substantial amount of experimental studies has found that eye movements during recall of unpleasant autobiographical memories decreased vividness or negative emotions, or both, associated with these emotions compared to recall only [35,67,68,69]. Furthermore, analogue studies have shown that other taxing tasks, like drawing a complex figure [70], playing Tetris [71], and mental arithmetic [72] all reduce image vividness or emotionality, or both. Gunter and Bodner [70] argue that the eye movement dual task has long-term effects because the nature of the memory trace remains unchanged but the meta-cognitive interpretations have been altered. Consequently, through eye movements (like in EMDR) and computer games like Tetris (which has recently translated into populations, e.g., Iyadurai et al. [73], Horsch et al. [74]), a considerable gain has been demonstrated with intrusive suicidal images. Thus, this may be a promising task to apply to intrusive suicidal images.

One possible hypothesis to account for the effects of the dual task is provided by the working memory model. The working memory is a multicomponent system that carries out higher-order cognitive functions (i.e., problem solving [75]). It consists of the central executive and two ‘buffer’ subsystems—the phonological loop (PL), and the Visuospatial Sketchpad (VSSP)—in which the central executive can allocate information to be held online for later use [76]. The PL stores verbal and auditory information, while the VSSP stores visuospatial information, and the latter is suggested to hold memories during EMDR sessions [67,77]. Images of unpleasant memories are held in the VSSP, and become less vivid when eye movements simultaneously use up processing resources in the VSSP. Controversy exists regarding what part of the working memory needs to be taxed. Engelhard and van den Hout [78] argue that taxing the central executive is sufficient, while others suggest that taxing the VSSP is most crucial for the effect [70]. Either way, there is an increased working memory load reducing the resources available. Thus, this working memory manipulation reduces the vividness of the memory and in turn decreases its emotionality.

One particular treatment using the effects of dual task is the EMDR Therapy. This is an evidence-based dual-task treatment for trauma-related intrusions [79], which is recommended across guidelines worldwide [80]. The patient retrieves and holds an image of the worst moment of their trauma in mind along with any associated negative cognitions and emotions. Concurrently, the therapist introduces an external stimulation (this often involves tracking the therapist’s finger moving from side to side, creating horizontal eye movements). Nowadays, therapists often use tools like a light bar to introduce this external stimulation. The set of eye movements are repeated until the memory no longer evokes stress as measured on the Subjective Units of Distress Scale [81]. Patients are instructed to allow any images and thoughts to enter and leave awareness, observing them but not trying to influence the process. It is hypothesized that these images and thoughts are part of the association-chain and have an important (subconscious) link to the intrusive memory. There has been controversy surrounding EMDR about whether eye movements added to the treatment’s effectiveness, or that it was a mere exposure effect that caused it to be effective. However, a recent meta-analysis found a significant positive effect of the eye movement component [82].

EMDR Therapy for PTSD has recently been applied effectively, easily and safely in severe psychiatric disorders, such as individuals with comorbid psychosis [83]. Using an eye-movement dual task with suicidal patients did not increase their suicidal risk, and appears to be a safe option for treatment [84]. Moreover, actively asking patients about their suicidality (i.e., through questionnaires, telephone calls, or interviews) did not increase their suicide risk. Similar results were found where displaying suicidal images did not increase the suicide risk of the participant [85]. Thus, specifically targeting patient’s suicidal intrusions should not be associated with an increased risk of suicide. Given the available evidence on dual tasks, as well as the known benefits of a treatment like EMDR, the treatment protocol discussed in this article proposes the use of EMDT with intrusive suicidal images. The EMDT add-on treatment uses the dual task component (image retrieval and an eye-movement task) to target negative suicidal intrusions. While EMDR Therapy has an additional explicit focus on reinstalling a positive cognition, EMDT solely focuses on reducing the vividness and emotional intensity of the intrusive mental image. Moreover, EMDR targets both positive and negative images, but EMDT in relation to suicide will focus solely on negative images.

## 4. Treatment of Intrusive Suicidal Images

Future-oriented images are located in the ‘prospective memory’ and can be stripped of their impact in the same way as flashbacks [35]. The working memory model implies that the past or future-oriented nature of vivid images is irrelevant to the effect of working memory taxing tasks such as eye-movements. Engelhard et al. [78] found that eye-movements indeed reduced vividness and emotionality of visual images about feared future events in a non-clinical sample. This suggests that EMDT could be a potential intervention focus to target future-oriented suicidal intrusions in suicidal individuals.

Based on the pre-suicidal syndrome, Escape Theory and Interpersonal-Psychological Theory, an EMDT intervention could target the suicidal intrusions that may indirectly affect the transition from suicidal ideation to suicidal behavior. Given the abundance of research evidence of dual tasks such as eye movements in other disorders, most notably PTSD, the approach is currently being tested experimentally and clinically in disorders like agoraphobia [86] and anxiety disorders [87]. Using suicidal intrusions as the target of the intervention may influence the underlying process of suicidal ideation. This process includes risk factors associated to suicide like hopelessness, desperation, entrapment, perceived burdensomeness, and wanting to escape the self. It could be hypothesized that when the images become less emotionally charged, the desire to act upon suicidal thoughts decreases. In turn, as the uncontrollable, vivid, suicidal intrusions diminish, their desire to escape them may decrease as well. There is an instant sense of relief, and the individual becomes able to shift their focus and attention to more positive non-suicide related thoughts.

In addition, targeting suicidal intrusions may also interfere with the individual’s ruminative thought processes. Rumination may be triggered and used as a coping strategy to try and avoid the suicidal intrusions. This escape mechanism is no longer needed and in turn decreases the level of rumination in the individual. Results of Petit’s study [88] show that suppression of suicidal thoughts may indeed be one of the mechanisms that contributes to the persistence of suicidal ideation. Approximately 450 cases of suicide each year are being treated for depression in the Dutch mental health care (extrapolated from Huisman et al. [89]). If targeting suicidal intrusions, as an intervention method is deemed effective, perhaps a substantial proportion of these suicides can be prevented. Implementation of this intervention in health care clinical settings, may reach the appropriate target audience and aid in reducing the number of suicides each year.

## 5. Treatment Protocol

The treatment protocol of EMDT treatment for suicidal intrusions (images and thoughts) focuses on taxing the working memory while retrieving suicidal images and thoughts. It consists of six sessions, each of approximately 1 h, over the course of three weeks. The intervention is aimed primarily at reducing the intensity and frequency of suicidal imagery and secondary to prevent suicidal behavior. The EMDT protocol has been developed in co-creation with patients and mental health professionals involved in mental health care for depressed patients with suicidal ideation, currently in treatment for depression or suicidality, or both. EMDT does not replace the usual care a suicidal patient needs (usually consisting of a combination of psychotherapy or medication, or both-), but it is an add-on intervention specifically targeted to reduce suicidal intrusions.

### 5.1. Introduction

Prior to the dual task, the patient needs to understand the concept of suicidal intrusions and how to identify their associated intrusive images. The patient and therapist have discussed the negative, emotional experience and content of the targeted suicidal intrusion in great detail. Once both therapist and patient feel confident about the established target suicidal intrusion, an introduction about EMDT will be provided. The therapist locates him- or herself in the appropriate position, so that the patient can have their full focus on the finger of the therapist.

### 5.2. Focus (Assessment)

During the assessment phase, the therapist tries to get the patient to be in the heavily emotionally charged state as they feel when experiencing the suicidal intrusions. This is essential, as the fear-network needs to be highly activated in order to generate any changes to the stimulus-aspects, meaning-aspects, and response-aspects associated to the memory representation of the intrusive suicidal image. The target image is selected and portrays the part of the suicidal intrusion that invokes maximum emotional tension.

### 5.3. Cognitive Domains

Similar to the EMDR procedure, there are five potential cognitive domains linked to the dysfunctional meaning of the suicidal intrusions. These are potential underlying concepts, which cause the suicidal thoughts and images to be uncontrollable and intrusive. First, responsibility, shame, and guilt are part of the cognitive domain in which the patient might blame themselves for certain (negative) life events. Second, control is an important cognitive domain, as they feel powerless or helpless and suicide seems to be the only solution. Third, self-worth is a cognitive domain in which the patient experiences strong negative self-conceptions. Fourth, safety is a potentially interesting cognitive domain as they experience a constant state of danger, and suicide seems to be the best or only option. Fifth, anger or revenge feelings, or both, towards others are a potential cognitive domain. 

### 5.4. Negative Cognition

The purpose of addressing the underlying maladaptive cognition is to figure out why this particular target image is intrusive and uncontrollable. The therapist asks the patient ‘why’ this specific image is so aversive (or positive) and what is says about the patient as a person. It is important to distinguish the emotional state from cognitions in this phase. For example, if the patient is feeling scared, their negative cognition could be: ‘I’m a coward’. The distress experienced with this image is measured using a ten-point Subjective Units of Distress Scale (SUDS).

### 5.5. Desensitization

It is emphasized that the focus of the patient does not have to stay on the initial target image the patient selected. Their mind is free to wander as it is hypothesized that any associative cognition that arises is important in the entire ‘associations-chain’ linked to their suicidal intrusion. The therapist does not initiate a conversation about the content of the associations that arise, but merely asks the patient to focus on the associations. The distress experienced with the target image is once again measured using the SUDS. Eye-movements are repeated until the score has diminished to 0.

### 5.6. Positive Closure

The patient is asked about what the most positive or worthwhile lesson or experience was during the session. The purpose of this positive closure is to have the patient leave with a powerful, positive feeling after the emotionally laden session.

### 5.7. Outcome Measures

We expect the EMDT-add on treatment to have an effect on the presence of suicidal intrusions, and various components of this suicidal intrusion. Most importantly, we are looking at a potential decrease in the frequency and intrusiveness of these suicidal ideas and mental imagery. Furthermore, the vividness, emotionality, and quality and content of the suicidal intrusion are evaluated as well. The Intrusion Interview [5] will measure all these components. Given that we are aiming to indirectly affect the possible transition from suicidal ideation to suicidal behavior, the Beck Suicidal Ideation Scale [90] is included. Moreover, we will keep track of the amount of suicide attempts over time. Other, secondary outcome measures include: severity of depression (Beck Depression Inventory-II (BDI-II); [91]), rumination (Rumination Response Scale (RRS); [92]), hopelessness (Beck Hopelessness Scale (BHS); [93]), and anxiety levels (Generalized Anxiety Disorder-7 (GAD-7); [94]). Finally, to touch upon the potential underlying working mechanisms of the EMDT-add on intervention, outcome measures such as the executive functioning ‘n-back task’ and rumination are considered. An instrument like the n-back task (see Braver and colleagues for a detailed description [95]) increases the working memory load, and is suggested to require executive processes. Given that eye movements performed during EMDT may tax the effect of EMDT as it may identify the underlying working mechanism of the intervention.

## 6. Conclusions

Repetitive intrusive suicidal ideas and mental imagery may potentially be an appropriate target to indirectly affect suicidal ideation and suicidal behavior. The cognitive effort of trying to stop the unwanted, vivid suicidal intrusions appears to have a counterproductive effect as it may inflate their frequency and intensity instead. Various experimental studies have found that eye movements during recall of the unpleasant images decreased their vividness and negative emotions. Other studies suggest that disrupting mental images in related ways reduces the frequency of their recurrence. Thus, an EMDT-ad on treatment targeting the suicidal intrusions may reduce their frequency and intensity. This may lead to an instant sense of relief, allowing the individual to focus on topics other than their suicide. Before EMDT may be used in clinical practice, empirical testing is required. We are therefore currently setting up a randomized controlled trial in which EMDT as an add-on intervention to usual care is tested against usual care alone in depressed patients with suicidal ideation and suicidal imagery.
